# Cigarette Smoking Alters the Expression of Circulating microRNAs and Its Potential Diagnostic Value in Female Lung Cancer Patients

**DOI:** 10.3390/biology10080793

**Published:** 2021-08-18

**Authors:** Eric Gustavo Ramírez-Salazar, Luis Vicente Gayosso-Gómez, Renata Baez-Saldaña, Ramcés Falfán-Valencia, Rogelio Pérez-Padilla, Anjarath L. Higuera-Iglesias, María E. Vázquez-Manríquez, Blanca Ortiz-Quintero

**Affiliations:** 1 Consejo Nacional de Ciencia y Tecnología (CONACYT), Instituto Nacional de Medicina Genómica, Mexico City 14610, Mexico; eramirez@inmegen.edu.mx; 2 Department of Research in Biochemistry, Research Unit, Instituto Nacional de Enfermedades Respiratorias Ismael Cosío Villegas, Mexico City 14080, Mexico; lvg1_1@hotmail.com; 3 Pneumology-Oncology Service, Instituto Nacional de Enfermedades Respiratorias, Mexico City 14080, Mexico; baezrd@gmail.com; 4 HLA Laboratory, Instituto Nacional de Enfermedades Respiratorias Ismael Cosío Villegas, Mexico City 14080, Mexico; rfalfanv@iner.gob.mx; 5 Department of Research in Tobacco and COPD, Instituto Nacional de Enfermedades Respiratorias Ismael Cosío Villegas, Mexico City 14080, Mexico; perezpad@gmail.com; 6 Department of Clinical Epidemiology Research, Instituto Nacional de Enfermedades Respiratorias Ismael Cosío Villegas, Mexico City 14080, Mexico; higuera.iglesias.anjarath@gmail.com; 7 Department of Pathology, Instituto Nacional de Enfermedades Respiratorias Ismael Cosío Villegas, Mexico City 14080, Mexico; euvazman@gmail.com

**Keywords:** lung adenocarcinoma, circulating microRNAs, cigarette smoking, diagnostic biomarker

## Abstract

**Simple Summary:**

In this study, we investigated whether circulating microRNA expression levels and their potential diagnostic value are affected by cigarette smoking in lung cancer patients and healthy participants. Our findings support that cigarette smoking affects the reliable identification of circulating miRNAs as diagnostic biomarkers in lung cancer and suggest a smoking-dependent pathogenic role of miR-133a-3p in smokers.

**Abstract:**

Cigarette smoking is a known risk factor for the development of lung cancer. We investigated whether circulating microRNA expression levels and their potential diagnostic value are affected by cigarette smoking in adenocarcinoma (AD) patients and healthy (H) participants. In total, 71 female AD patients and 91 H individuals were recruited, including 42 AD never-smokers (AD/CS−), 29 AD smokers (AD/CS+), 54 H never-smokers (H/CS−), and 37 H smokers (H/CS+). PCR array (754 microRNAs) and qPCR were performed on sera from the discovery and validation cohorts, respectively. The expression levels of miR-532-5p, miR-25-3p, and miR-133a-3p were significantly higher in adenocarcinoma patients than in healthy participants, independent of their smoking status. Multivariate analysis showed that levels of miR-133a-3p were independently associated with smoking. ROC analysis showed that only miR-532-5p discriminated AD patients from H controls (AUC: 0.745). However, when making comparisons according to cigarette smoking status, miR-532-5p discriminated AD/CS− patients from H/CS− controls with a higher AUC (AUC:0.762); miR-25-3p discriminated AD/CS+ patients from H/CS+ controls (AUC: 0.779), and miR-133a discriminated AD/CS+ patients from H/CS+ controls with the highest AUC of 0.935. Cancer and lung-cancer-enriched pathways were significantly associated with the three miRNAs; in addition, nicotinate/nicotinamide metabolism, inflammation, and pulmonary hypertension were associated with miR-133a-3p. Our findings highlight how cigarette smoking affects the reliable identification of circulating miRNAs as diagnostic biomarkers in lung cancer and suggest a smoking-dependent pathogenic role of miR-133a-3p in smokers.

## 1. Introduction

Lung cancer remains the leading cause of cancer-related deaths worldwide, with one of the lowest 5-year survival rates, 15–20% [1]. The high mortality in lung cancer patients has been largely attributed to diagnosis in advanced stages of the disease when most treatments are no longer effective; therefore, the search for reliable and accurate diagnostic biomarkers has become a field of high relevance.

Among environmental factors, cigarette smoking is the most important risk factor for the development of lung cancer [2,3]. Studies have shown that cigarette smokers have a 10–25-fold greater risk of dying from lung cancer than non-smokers [3]. In addition, it is known that cigarette smoke induces genetic and molecular alterations in bronchial epithelial cells that may cause lung carcinogenesis [4].

MicroRNAs (miRNAs) are short noncoding RNAs that regulate the gene expression by recognizing target mRNAs and subsequently inducing the inhibition of translation or transcript degradation. miRNAs participate in the epigenetic regulation of virtually all cellular processes studied under normal and disease conditions [5]. They are detected in biological samples, such as tissue, peripheral blood, and other body fluids in a highly stable and quantifiable form, and their aberrant expressions have been associated with the presence of multiple cancers, including lung cancer [6,7,8]; therefore, miRNAs have a potential clinical value as diagnostic biomarkers. In particular, circulating miRNAs in serum, plasma, and whole peripheral blood have been greatly studied as non-invasive biomarkers for lung cancer diagnosis [7], which may improve the diagnosis efficiency as an alternative to undergoing lung tissue biopsy. On the other hand, miRNAs expression can be affected by cigarette smoking. Cigarette smoking can lead to the deregulation of the global miRNAs in lung tissues [9], and in alveolar macrophages, as well as in the bronchial alveolar epithelium from smokers [10,11]. Moreover, lung cancer cell lines exposed to cigarette smoke express altered miRNAs which are associated with mechanisms of carcinogenesis [12,13]. Regarding circulating miRNAs, three studies using small cohorts reported that cigarette smoking is associated with alterations in the expression of circulating miRNAs in the plasma of healthy individuals [14,15,16], whereas a recent study using a large cohort reported a smoking-associated miRNA signature in whole blood from healthy subjects that could be associated with inflammation [17]. However, despite the known association between cigarette smoking and lung cancer, little has been reported regarding the effect of smoking on the expression levels of circulating miRNAs in lung cancer and how it affects the identification of potential candidate miRNAs as non-invasive biomarkers of this disease. In the present study, we investigated whether circulating microRNA expression levels and their potential diagnostic value are affected by cigarette smoking in lung cancer patients and healthy participants. We also searched for the potential biological relevance of smoking-related miRNAs in lung pathogenesis.

## 2. Materials and Methods

### 2.1. Study Population and Design

The participants in this study were recruited under an Institutional Research protocol approved by the Ethics and Science Committee at the Instituto Nacional de Enfermedades Respiratorias Ismael Cosio Villegas (INER, Mexico City, Mexico), protocol code B30-15. Written informed consent was obtained from all the participants. Patients with histological diagnosis of primary non-small-cell lung cancer (NSCLC) subtype adenocarcinoma (AD) were recruited from the Pneumology-Oncology Service at the INER. The patients included in this study did not receive adjuvant or radiation therapy prior to the serum sample collection. Lung cancer patients were selected based on the availability of serum samples and complete demographic, cigarette smoking (CS) history, and histopathological information. Exclusion criteria included diagnosis of asthma, chronic obstructive pulmonary disease (COPD), associated cancer, HIV infection, or diagnosis of respiratory tract infection in the preceding 4 weeks of sample collection. For this study, only women older than 50 years old were included. Gender-matched control serum samples were obtained from healthy never-smoker (H/CS−), and smoker (H/CS+) women recruited from blood donation and smoking cessation campaigns at INER, Mexico City.

AD cases (*n* = 71) were randomly distributed into discovery (*n* = 30) and validation (*n* = 41) sets. Controls (*n* = 91) were distributed into discovery (*n* = 30) and validation (*n* = 61) sets. The discovery set consisted of 15 AD never-smoker (AD/CS−) patients, 15 AD smoker (AD/CS+) patients, 15 healthy never-smoker (H/CS−) participants, and 15 healthy smoker (H/CS+) participants. The validation set consisted of 27 AD/CS−, 14 AD/CS+, 39 H/CS−, and 22 H/CS+ participants.

In the discovery phase, the miRNA expression profile (754 human miRNAs) was assessed in the serum samples from AD and healthy control participants using quantitative polymerase chain reaction (PCR) array technology. The miRNAs differentially expressed in AD patients compared with those in controls were identified according to cigarette smoking status (*p* < 0.05 and log_2_ Fold Change > 2). For the validation phase, the levels of individual candidate miRNAs were validated by quantitative polymerase chain reactions (qPCR) in the validation set.

### 2.2. Cigarette Smoking Assessment

A questionnaire was applied at baseline to obtain a detailed history of cigarette smoking. Never-smokers were individuals never exposed to cigarette smoke actively or passively. Smokers with a cigarette smoking index of ≥10 pack-years (calculated by multiplying the number of cigarettes per day by the number of years spent smoking divided by 20 cigarettes in one pack) were included in this study.

### 2.3. Serum Samples

Blood samples were drawn from the cubital vein and collected into BD Vacutainer SST blood collection tubes without anticoagulant and with a clot separator gel (REF 368159). After 30 min, sera were separated by centrifugation at 1200× *g* for 15 min (room temperature), aliquoted, and stored at −80 °C until use.

### 2.4. RNA Isolation

The extraction of RNA from 200 μL serum samples was performed using the miRNeasy Serum/Plasma Kit (cat. 217184, QIAGEN, Hilden, Germany) according to the manufacturer’s instructions. Synthetic Caenorhabditis elegans (C. elegans) miRNA cel-miR-39 was added to serum samples during the extraction step (validation cohort). This enabled normalization for any nonspecific losses incurred during miRNA purification [18]. The RNA concentration and purity were assessed using a Nanodrop ND-2000 spectrophotometer (Thermo Fisher Scientific, Waltham, MA, USA), and RNA samples were stored at −80 °C until use. We assessed hemolysis in all serum samples by measuring the absorbance of hemoglobin at 414 nm with a Nanodrop ND-2000 spectrophotometer (Thermo Fisher Scientific, Waltham, MA, USA) and in a randomly selected 50% of the RNA samples by measuring the delta Ct value of miR-23a and miR-451 using qPCR [19].

### 2.5. MicroRNA Expression Profiling

For the discovery phase, we determined the serum miRNA expression profile of three RNA pools (five samples per pool) of each study group using the TaqMan Human MicroRNA Array Panel v3.0 (Applied Biosystems, CA, USA), which includes cards A and B in a 384-well format and probes for 754 human miRNAs. The quantification procedure was performed according to the manufacturer’s instructions. Briefly, 30 ng of pooled RNA was reverse transcribed (RT) using the Megaplex RT stem-loop primer pools A and B (Applied Biosystems, Foster City, CA, USA). Subsequently, Megaplex RT products were pre-amplified using Megaplex PreAmp Primers (Pool A and B) and TaqMan PreAmp Master Mix (Applied Biosystems). Real-time PCR reactions were performed on the 7900 HT (Applied Biosystems) with the recommended cycling conditions. The differential expression of miRNAs between study groups was assessed using the Expression Suite Software v1.0.3 (Life Technologies). Data were normalized using the mean of the expression value (Ct) of all expressed miRNAs on the plate [20]. Only Raw Ct values lower than 35 were considered for analysis. The levels of miRNAs that showed values of *p* < 0.05 and a log2 foldchange > 2 in AD patients compared with those in controls were considered differentially expressed.

### 2.6. Validation by qPCR

For the validation phase, the levels of selected candidate miRNAs identified in the discovery phase were quantified in individual serum samples using TaqMan Advanced miRNA Assays (Thermo Fisher Scientific) on the Step-One-Plus Real-Time PCR System (Applied Biosystems). We selected arbitrarily the five miRNAs (miR-532-5p, miR-25-3p, miR-133a-3p, miR-200c-3p, and miR-29c-3p) with the most significantly differential expression in AD/CS− and AD/CS+ patients. All reactions were performed in triplicate. Only miRNAs with raw Ct values lower than 35 were considered for analysis. qPCR data were normalized using the mean of the exogenous spike-in miRNA cel-miR-39-3p (Assay ID 478293_mir) and the mean of the endogenous reference miRNA miR-339-3p (Assay ID 478325_mir), which was previously selected as the most stable normalizer by the NormFinder software [21]. The 2 − ΔCt method was used to calculate the relative expression quantities (RQ) of miRNAs levels. RQ = 2 − ΔCt, ΔCt = Average Mean Ct miRNA—Average Mean Ct normalizers.

### 2.7. Statistical Analysis

Continuous variables are presented as the mean (standard deviation) in normally distributed data or median (interquartile range) in variables without a normal distribution (Shapiro–Wilk test). Comparisons between continuous variables in multiple groups were performed using one-way ANOVA and Tukey’s post hoc test in normally distributed data (parametric data) and the Kruskal–Wallis test with Dunn’s post hoc test in variables without normal distribution (nonparametric data) followed by Mann–Whitney U test for pairwise comparisons. The chi-square test was used to compare the categorical variables between groups. Spearman’s rank test was used to test correlations between continuous variables. Univariate linear regression analysis followed by multivariate linear regression analysis was performed to adjust for potential cofounders and to determinate an association between miRNA levels and clinical and demographic variables in AD patients. Variables with a *p* < 0.10 in the univariate analysis were tested in the multivariate models. Two models of logistic regression analysis were tested: (1) using comorbidities and epidemiological factors (including the smoking status and pack/years) as the independent variables and the miRNA expression levels as the dependent variables; (2) using comorbidities, epidemiological factors, and miRNA expression levels as the independent variables and the smoking status (or pack/years) as the dependent variable. Receiver operating characteristics (ROC) curve analysis were used to determinate the diagnostic accuracy values of miRNAs for distinguishing between AD patients and H subjects and the effect of cigarette smoking status. Additionally, we performed a binary logistic regression and the ROC curve analysis to test a model for the diagnostic value of the combinations of the three selected miRNAs. Analysis was performed using SPSS software package version 26.0 (SPSS, Chicago, IL, USA), and graphs were constructed using GraphPad Prism version 8.

### 2.8. MiRNA Target Genes and Pathways Enrichment Analysis

We used the miRNet platform (http://mirnet.ca, accessed on 10 June 2021) to obtain the experimentally validated target genes of the candidate miRNAs (miRTarBase v8.0 and TarBase v8.0 databases) to perform an enrichment pathway analysis (Kyoto Encyclopedia of Genes and Genomes database or KEGG) and miRNA-to-disease interaction analysis (DisGeNET database). Pathways with significant enrichment have *p* < 0.01 (KEGG) and *p* < 0.005 (DisGeNET). Enriched pathways showing statistical significance were subjected to further molecular analysis and interactome network construction. The resulting networks were exported to Cytoscape v3.1.0, with the plug-in centiscape, for visualization (http://cytoscape.org/, accessed on 10 June 2021). In the networks, the size of the nodes corresponds to the number of interactions between nodes.

## 3. Results

### 3.1. Baseline Characteristics of the Study Population

The clinical and demographic characteristics of AD patients and healthy participants according to their cigarette smoking status are shown in Table 1. A total of 71 AD patients and 91 healthy controls were recruited, which included 42 never-smoker AD (AD/CS−) patients, 29 smoker AD (AD/CS+) patients, 54 never-smoker healthy (H/CS−) controls, and 37 smoker healthy (H/CS+) controls. Among smokers, healthy (H/CS+) controls had significantly higher packs/year than AD/CS+ patients in both the discovery and validation cohorts. There were no significant differences in age, body mass index (BMI), or obesity among the participants. There were no significant differences in the diagnosis of diabetes mellitus and systemic hypertension among AD patients. However, AD/CS− and AD/CS+ patients showed significant differences in diagnosis of cardiac disease in the validation cohort.

### 3.2. Expression Profiling of miRNAs in the Sera of AD Patients According to Their Cigarette Smoking Status

**Figure 1:**
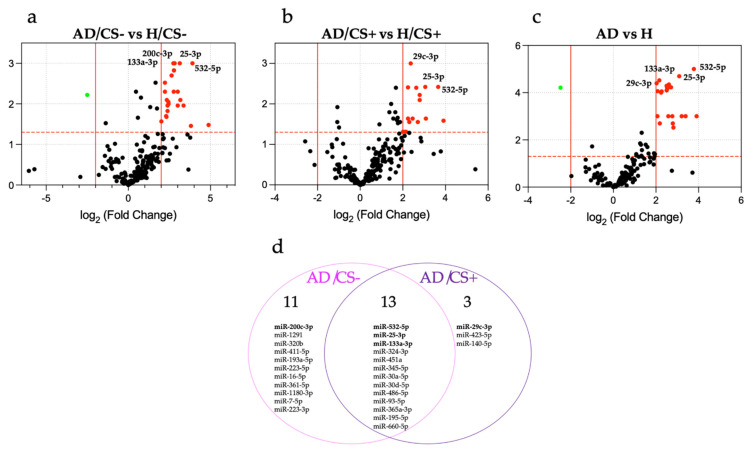
Analysis of the differentially expressed miRNAs in AD patients according to cigarette-smoking status, identified in the discovery phase. Volcano plots of the miRNA profiles (**a**) AD/CS− compared with H/CS−, (**b**) AD/CS+ compared with H/CS+, (**c**) AD compared with H. The red dots represent differential upregulation of miRNAs (*p* < 0.05 and log2Fold Change > 2), and the green dots represent differential downregulation of miRNAs (*p* < 0.05 and log2Fold Change < −2). (**d**) Venn diagram analysis of the differentially expressed miRNAs in AD patients according to cigarette-smoking status. AD, adenocarcinoma; CS, cigarette smoking; H, healthy controls.

In the discovery phase, 24 miRNAs were found to be differentially expressed in AD/CS− patients compared with H/CS− controls (Figure 1a and Table S1), whereas 16 miRNAs were differentially expressed in AD/CS+ patients compared with H/CS+ controls (Figure 1b and Table S2). In addition, 24 miRNAs were differentially expressed in AD patients compared with H controls, regardless of their cigarette smoking status (Figure 1c and Table S3). Among these miRNAs, 12 miRNAs were common in both AD/CS− and AD/CS+ patients; 12 miRNAs were found exclusively in AD/CS− patients, and 3 miRNAs were found exclusively in AD/CS+ patients (Figure 1d).

Table S4 shows the complete results of the analysis of differentially expressed miRNAs in the screening phase.

### 3.3. Validation of Differentially Expressed miRNAs by qPCR

**Figure 2:**
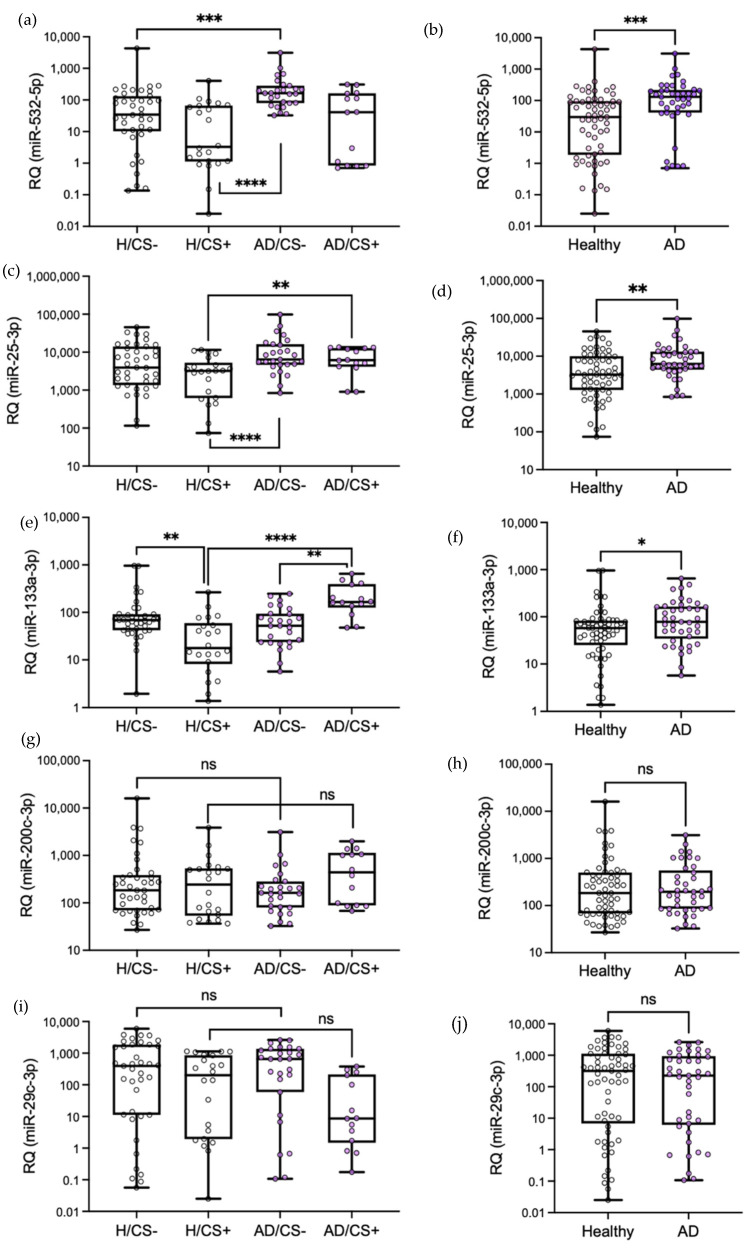
Validation of differentially expressed miRNAs in AD patients compared to healthy controls according to (**a**–**e**) cigarette-smoking status, and (**f**–**j**) study group. Statistically significant differences were indicated as * *p* < 0.05, ** *p* < 0.01, *** *p* < 0.001, **** *p* < 0.0001, and ns = no statistical significance, according to Kruskal–Wallis test with Dunn’s post hoc test, followed by Mann–Whitney U test for pairwise comparisons. RQ, relative expression; AD, adenocarcinoma; CS, cigarette smoking; H, healthy controls. RQ values are presented in logarithm base 10 scale.

We selected five candidate miRNAs (miR-532-5p, miR-25-3p, miR-133a-3p, miR-200c-3p, and miR-29c-3p) with the most significantly differential expression in AD/CS− and AD/CS+ patients (Tables S1 and S2) for validation in individual serum samples using qPCR. Three of them were common in both study groups (miR-532-5p, miR-25-3p, and miR-133a-3p; Figure 1d); one was found only in AD/CS− patients (miR-200-3p, Figure 1d), and one was found only in AD/CS+ patients (miR-29c-3p, Figure 1d). These five selected miRNAs were also found differentially expressed in AD patients compared with controls regardless of their smoking status (Table S3). The results confirmed the significantly higher expression of miR-532-5p, miR-25-3p, and miR-133a-3p in AD patients compared with healthy controls, regardless of their smoking status in the validation cohort (*p* = 0.0003, *p* = 0.003, and *p* = 0.029, respectively), whereas no statistically significant differences were found for miR-200c-3p or miR-29c-3p (*p* > 0.05) (Figure 2f–j). Regarding cigarette smoking status, we observed a significantly higher expression of miR-532-5p in AD/CS− patients than in H/CS− and H/CS+ controls (*p* = 0.0002 and *p* < 0.0001, respectively) but not in AD/CS+ patients when compared with healthy controls (*p* > 0.05) (Figure 2a). We also observed the significantly higher expression of miR-25-3p in AD/CS+ patients than in H/CS+ controls (*p* = 0.004) and in AD/CS− patients when compared with H/CS+ controls (*p* = 0.0006) but not in AD/CS− patients when compared with H/CS− controls (*p* > 0.05) (Figure 2b). Moreover, we noted the significantly higher expression of miR-133a-3p in AD/CS+ patients than in H/CS+ controls (*p* < 0.0001) but not in AD/CS− patients when compared with all control groups (Figure 2c). Additionally, we found that the expression of miR-133a-3p was significantly lower in healthy smokers (H/CS+) than in never-smokers (H/CS−) (*p* = 0.0005). Conversely, miR-133a-3p was significantly higher in AD smoker (AD/CS+) patients than in AD never-smoker (AD/CS−) patients (*p* = 0.0002), which may indicate a potential positive association between miR-133a and smoking in AD patients, but there was an inverse association in healthy participants. This inverse association may contribute to the observed significantly higher *p*-value (**** *p* < 0.0001) in AD/CS+ patients than in H/CS+ controls (Figure 2c) and to the less significant *p*-value (* *p* = 0.029) in AD patients than in H controls (Figure 2h), which included both smoker and never-smoker healthy participants. Regarding miR-200c-3p and miR-29c-3p, no significant differences were found in AD/CS− or AD/CS+ patients when compared with all healthy control groups (*p* > 0.05) (Figure 2d,e), despite significant differences found in the PCR array analysis.

### 3.4. The Potential Diagnostic Value of the Differentially Expressed miRNAs in AD Patients, miR-532-5p, miR-25-3p, and miR-133a-3p, Is Affected by Cigarette Smoking Status

**Figure 3:**
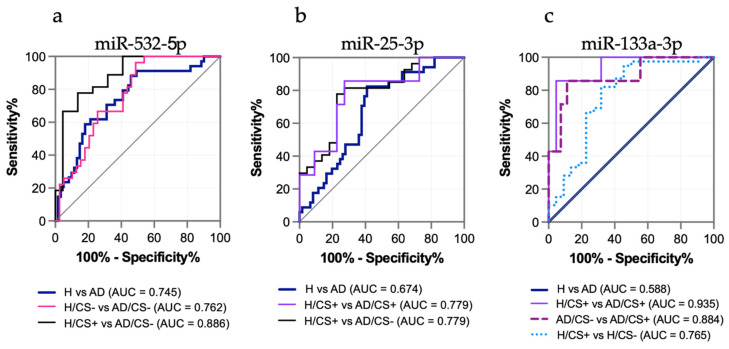
Receiver operator characteristic (ROC) curve analysis of (**a**) circulating miR-532-5p, (**b**) miR-25-3p and (**c**) miR-133a-3p according to the cigarette-smoking (CS) status of adenocarcinoma (AD) patients and healthy (H) participants.

We performed receiver operator characteristic (ROC) curve analysis to evaluate the diagnostic value of the three candidate miRNAs and the potential effect of cigarette smoking status. When the groups were compared regardless of smoking status (H versus AD), only miR-532-5p discriminated AD patients from healthy controls with an area under the curve (AUC) higher than 0.7 (AUC: 0.745, *p* < 0.0001), whereas miR-25-3p showed a low AUC value (AUC: 0.674, *p* = 0.005), and miR-133a-3p did not achieve any significant discriminatory capacity (AUC: 0.588, *p* = 0.158) (Figure 3a–c). When groups were compared according to their cigarette smoking status, miR-532-5p discriminated AD/CS− patients from H/CS− controls with a higher AUC of 0.762 (*p* < 0.0001) and AD/CS− patients from H/CS+ controls with an AUC of 0.886 (*p* < 0.0001) (Figure 3a). Among them, the best sensitivity of 77.8% and specificity of 81.8% were achieved for AD/CS− patients versus H/CS+ controls (Table 2). miR-25-3p displayed a better diagnostic value when AD/CS+ patients were compared with H/CS+ controls (AUC: 0.779, sensitivity: 81.5%, and specificity: 72.7%) and AD/CS− patients with H/CS+ controls (AUC: 0.779, sensitivity: 85.7%, and specificity: 72.7%) (Figure 3b). Although miR-133a-3p did not show significant diagnostic value for AD patients when compared with healthy controls, miR-133a displayed a high AUC of 0.935 with a sensitivity of 85.7% and specificity of 95.5% when AD/CS+ patients were compared with H/CS+ controls (Figure 3c and Table 2), showing a striking discriminatory capacity for AD patients among smokers. In accordance with the results from qPCR validation, levels of miR-133a-3p were also able to discriminate AD/CS+ patients from AD/CS− patients (AUC: 0.884, sensitivity: 85.7%, and specificity: 88.9%) and H/CS+ controls from H/CS− controls (AUC: 0.765, sensitivity: 82.1, and specificity: 68.2) (Figure 3c and Table 2).

Additionally, we performed a logistic regression analysis to investigate whether the combination of three or two candidate miRNAs can improve their diagnostic value. The results indicated that the sensitivity and specificity values did not improve substantially when three or two miRNAs were combined for the diagnosis of AD vs. H (Table 2 and Table S5). However, the sensitivity value improved from 85.7% to 100% when the combination of miR-25-3p and miR-133a-3p was used for the diagnosis of AD smokers (AD/CS+ vs. H/CS+), although the specificity decreased from 95.5% to 86.6% (Table 2).

### 3.5. Levels of miR-133a-3p Were Independently Associated with Cigarette Smoking in AD Patients

A Spearman correlation analysis was performed to determinate the potential relationship between the levels of differentially expressed miRNAs and the continuous variables in AD patients and healthy participants, including pack/years for cigarette smoking (Table 3). Levels of miR-532-5p showed a moderate negative correlation with pack/years in AD patients and a weak negative correlation in healthy participants, in addition to a moderate positive correlation with age in healthy participants. There was a moderate positive correlation between miR-25-3p levels and age in healthy participants. In contrast, the levels of miR-133a-3p showed a moderate positive correlation with the pack/years in AD patients, but a negative correlation in healthy participants, in addition to a positive correlation with age in AD patients.

However, considering all the comorbidities and epidemiological factors (independent variables) that differed according to the miRNA levels (dependent variables) in AD patients in the univariate analysis, the pack/years and hypertension were independently associated with the levels of miR-133a-3p in the multivariate logistic regression analysis (Table 4). There were no associations with miR-532-5p or miR-25-3p as dependent variables (Table S6). In addition, discrimination of AD/CS+ patients from AD/CS− patients (ID groups) was independently associated with the miR-133a-3p levels (Table 4).

Considering all the comorbidities, epidemiological factors and miRNA levels as independent variables and the smoking status or pack/years as dependent variables in the univariate analysis, only the levels of miR-133a-3p were independently associated with smoking and pack/years (Table 5 and Table S7).

### 3.6. Pathway Enrichment Analysis

**Figure 4:**
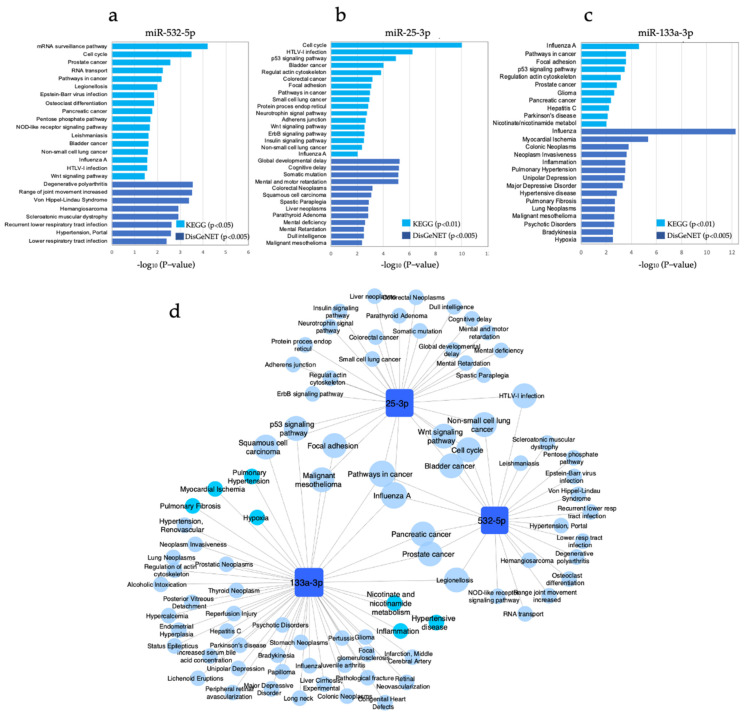
Pathway enrichment analysis of miR-532-5p, miR-25-3p and miR-133a-3p. (**a**–**c**) The figure shows the top significantly enriched pathways (KEGG, *p* < 0.01; and DisGeNET, *p* < 0.005) regulated by experimentally validated genes targeted by miR-532-5p, miR-25-3p and miR-133a-3p. (**d**) The network of the miRNAs and their significantly enriched pathways. The miRNAs are displayed as blue squares, and pathways are displayed as light blue circles. The size corresponds to the number of interactions between pathways and the miRNAs. Pathways associated with inflammation and smoking-associated diseases are highlighted with bright blue color. Cytoscape v3.1.0 was used for network visualization.

We further investigated the molecular pathways regulated by genes targeted by miR-532-5p, miR-25-3p, and miR-133a-3p. We identified the experimentally validated target genes using TarBase and mirTarbase and performed enriched pathways analysis (miRNet) based on the KEGG and DisGeNET databases. Figure 4 shows the top enriched pathways associated with cancer mechanisms among others (KEGG, *p* < 0.01; DisGeNET, *p* < 0.005), while Table S8 shows the complete list of enriched pathways. The analysis revealed statistically significant enrichment pathways associated with cancer and lung cancer for the three miRNAs (Figure 4a,b). Pathways in cancer, p53 signaling, lung neoplasm, non-small-cell lung cancer, cell cycle, and Wnt signaling pathways were among the enriched pathways. Notably, nicotinate and nicotinamide metabolism, inflammation, pulmonary hypertension, pulmonary fibrosis, myocardial ischemia, and hypoxia were enriched pathways regulated by genes targeted by miR-133a-3p, which was independently associated with cigarette smoking (Figure 4c,d). Among the cancer-related pathways, pathways in cancer was common for the three miRNAs; whereas non-small cell lung cancer, Wnt signaling pathway, cell cycle, and bladder cancer were common for miR-532-5p and miR-25-3p; p53 signaling pathway, focal adhesion, squamous cell carcinoma, and malignant mesothelioma were common for miR-25-3p and miR-133a-3p; and pancreatic cancer, and prostate cancer were common for miR-133a-3p and miR-532-5p (Figure 4d).

**Figure 5:**
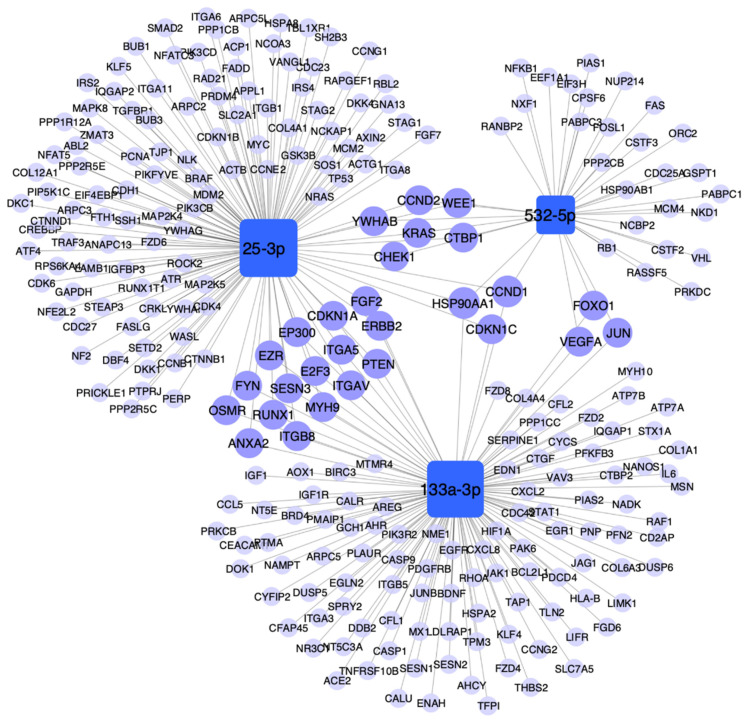
Network of miR-532-5p, miR-25-3p, and miR-133a-3p with their experimentally validated target genes. Figure shows the genes from selected pathways associated with cancer, lung cancer, inflammation, and smoking-associated diseases (*p* < 0.01, KEGG database; and *p* < 0.005 DisGeNET database). miRNAs are displayed as blue squares, and target genes are displayed as light purple circles. The genes shared by the miRNAs are shown as dark purple circles. The size corresponds to the number of interactions between genes and the miRNAs. Cytoscape v3.1.0 was used for network visualization.

We constructed a network to visualize the molecular interactions between the miRNAs and their experimentally validated target genes that regulate the identified pathways associated with cancer, lung cancer, inflammation, and smoking-associated diseases (Figure 5). This analysis showed that three genes were common targets for the three miRNAs: cyclin D1 (CCND1), cyclin dependent kinase inhibitor 1C (CDKN1C), and heat shock protein 90 alpha family class A member 1 (HSP90AA). All these target genes were involved in carcinogenesis mechanisms (Table S9). Other common target genes were vascular endothelial growth factor A (VEGFA), forkhead box 01 (FOX01), and jun proto-oncogene (JUN) for miR-532-5p and miR-133a-3p, which were associated with cancer and mechanisms related to inflammation, hypertensive disease, and myocardial ischemia (VEGFA and JUN). miR-25-3p and miR-133a-3p shared genes involved in cancer-related pathways such as cyclin dependent kinase inhibitor 1A (CDKN1A), E1A binding protein p300 (EP300), Erb-b2 receptor tyrosine kinase 2 (ERBB2), myosin heavy chain 9 (MYH9), integrin subunit alpha V (ITGAV), phosphatase and tensin homolog (PTEN), heat shock protein 90 alpha family class a member 1 (HSP90AA), frizzled class receptor 6 (FZD6), among others (Table S6).

**Figure 6:**
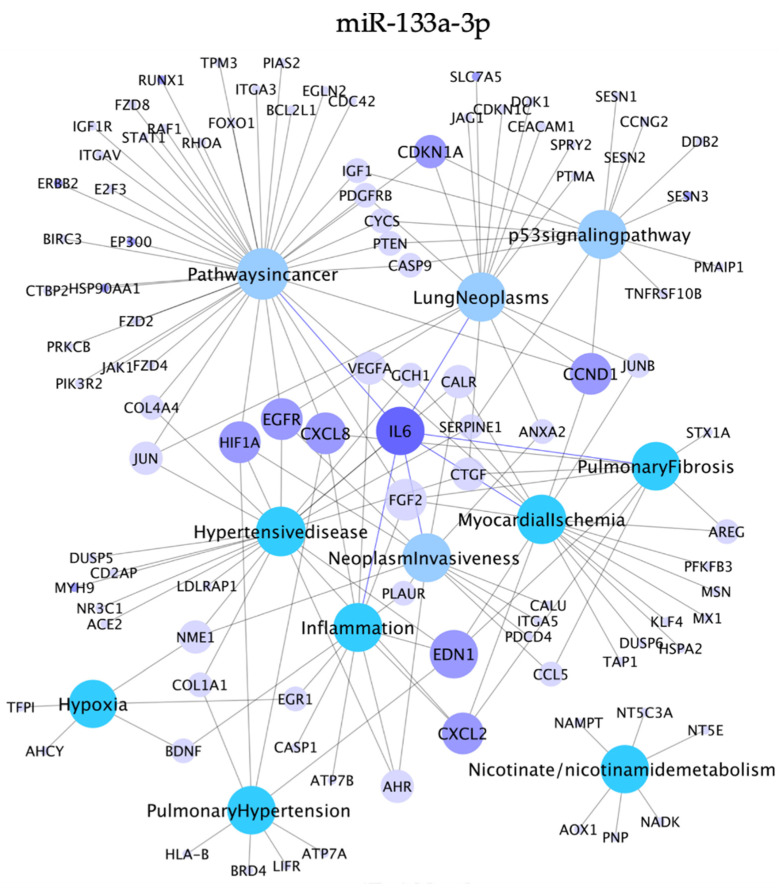
Network of the miR-133a-3p experimentally validated target genes and their enriched pathways. Figure shows the miR-133a-3p target genes that regulate selected pathways associated with cancer, inflammation, and smoking-associated diseases (*p* < 0.01, KEGG database; and *p* < 0.005 DisGeNET database). The target genes are displayed as light purple circles, and enriched pathways are displayed as circles of various colors. The size corresponds to the number of interactions between genes and pathways. The genes shared by more than two miRNAs (indicated in Figure 5) are shown as dark purple circles. Cytoscape v3.1.0 was used for network visualization.

To further analyze the potential biological role of the smoking-associated miR-133a-3p, we constructed a network with its experimentally validated target genes and their enriched pathways associated with cancer, inflammation, and smoking-associated diseases (Figure 6). The analysis showed highly interconnected target genes that should be further analyzed to elucidate the pathogenic mechanisms of non-cancerous smoking-associated diseases, such as Endothelin 1 (EDN1), which was associated with inflammation, pulmonary hypertension, pulmonary fibrosis, myocardial ischemia, and hypertensive disease (Figure 6). In addition, C-X-C Motif Chemokine Ligand 2 (CXCL2) was associated with hypertensive disease, inflammation, myocardial ischemia, and pulmonary fibrosis. On the other hand, IL6 appeared to be a link mechanism between cancer and inflammatory pathogenic mechanisms because it was found associated with lung inflammation, lung fibrosis, hypertensive disease, myocardial ischemia, pathways in cancer, lung neoplasms, and neoplasm invasiveness. Furthermore, the target genes CDKN1A and CCND1 may be involved in mechanisms that lead to cancer disease because they were associated with pathways in cancer, p53 signaling pathway, and lung neoplasms.

## 4. Discussion

We conducted a case–control study of lung adenocarcinoma cancer female patients and healthy female participants, smokers, and never-smokers, and found that cigarette smoking affects the expression patterns and reliable identification of circulating miRNAs as diagnostic biomarkers in lung cancer. Our findings also suggest that miR-532-5p, miR-25-3p, and miR-133a-3p are associated with mechanisms of lung carcinogenesis and reveals a potential smoking-dependent pathogenic role of miR-133a-3p in smokers.

We first performed a wide miRNA profiling on the sera of female AD and gender-matched healthy participants and validated selected miRNAs with quantitative qPCR as a classic strategy that led to the identification of three differentially expressed miRNAs in AD patients compared with healthy participants. Among the three candidate miRNAs, only miR-532-5p showed a potential diagnostic value for AD according to the ROC analysis. However, when we analyzed the study groups according to their smoking status, we first observed that differentially expressed miRNAs showed lower *p*-values for stratified groups than for the AD versus H groups (Figure 2), which were notably lower for miR-133a-3p when AD/CS+ patients were compared with H/CS+ controls (Figure 2c,h). This analysis also revealed that miR-133a-3p was significantly upregulated in AD/CS+ patients when compared with AD/CS− patients, but it was downregulated in H/CS+ participants when compared with H/CS− participants, indicating a potential opposite association with smoking. The positive association of smoking with miR-133a-3p was later confirmed in the multivariate analysis for AD, disclosing a smoking-associated miRNA in lung adenocarcinoma. Moreover, the stratified analysis showed that diagnostic values improved for the three miRNAs (Figure 3 and Table 2), with the best diagnostic value for miR-133a-3p (AD/CS+ versus H/CS+), even when this miRNA was unable to discriminate AD patients from H controls (Table 2). Therefore, in addition to affecting the expression level of circulating miRNAs, cigarette smoking affects the identification of miRNAs as potential biomarkers for the diagnosis of lung cancer.

We further investigated the potential biological relevance of these three miRNAs by performing enrichment pathway analysis of their experimentally validated target genes, and they all were associated with several mechanisms of carcinogenesis and lung cancer, such as pathways in cancer, p53 signaling pathway, cell cycle, Wnt signaling pathway, NSCLC, and lung neoplasms. Notably, smoking-associated miR-133a-3p also showed a significant association with nicotine/nicotinamide metabolism and mechanisms linked to smoking-related diseases, such as inflammation, pulmonary hypertension, pulmonary fibrosis, hypertensive disease, myocardial ischemia, and lung neoplasm, supporting the notion of a smoking-dependent pathogenic role of miR-133a-3p in smokers. miR-133a-3p may have a key role in mechanisms that lead to either cancer (target genes CDKN1A, and CCND1) or non-cancerous (target gene EDN1, and CXCL2) conditions secondary to smoking. Dysregulation of miR-133a-3p may also have an accumulative harming effect that may lead to either one or both of cancer and non-cancerous conditions (target genes IL6, and FGF2). Further investigations should be performed to elucidate the biological role of this circulating miRNA.

Few studies have reported alterations of circulating miRNAs in the plasma of smokers compared with that of non-smokers in small cohorts of healthy individuals [14,15,16], and one study examined the whole blood of smokers and non-smokers in a large cohort of healthy individuals [17]; however, our results (miR-133a-3p downregulated in smokers) do not show an overlap with the miRNAs identified in those previous studies. This could be due to the different source of miRNAs (plasma [14], plasma vesicles [16], or whole blood [17], instead of serum), which miRNAs were investigated (11 immune-/cancer-related miRNAs [15] or 84 cardiovascular disease-related miRNAs [16] instead of 754 screened miRNAs), or even the age of the participants (20-year-old [16], instead of >50-year-old individuals).

Kryczka et al. [22] reported that they did not find a statistically significant correlation between serum miRNA and the smoking history of 31 non-small-cell lung cancer (NSCLC) patients when compared with 21 control subjects; however, they investigated only five previously selected cancer-related miRNAs (miR-23a, miR-361, miR-1228, and miR-let7i), which may not be affected by smoking.

On the other hand, Huang et al. [23] analyzed the serum miRNAs in smokers versus non-smokers in lung cancer patients and healthy subjects and reported that let-7i-3p and miR-154-5p were downregulated in lung cancer smokers compared with healthy non-smokers, and in lung cancer smokers compared with lung cancer non-smokers. These results are also not in accordance with ours. The main differences were an all-male study population, the use of a microarray platform, and that lung cancer cases were a mixture of adenocarcinoma and squamous cell carcinoma. The latter may contribute to the lack of concordance since miRNAs are differentially expressed between these histological subtypes in tissue samples [24,25], and perhaps in blood circulation. The use of different platforms for miRNA quantification is also one reason for the heterogenous results reported by published studies due to differences in sensitivity and specificity among them [7,26].

Our three discovered miRNAs, miR-532-5p, miR-25-3p, and miR-133a-3p, have been identified as potential biomarkers for various types of tumors and linked to cancer-related mechanisms in the literature [27,28,29,30,31]. Because miR-133a-3p was associated with smoking status and showed the best diagnostic value for AD smokers, we further investigated it. miR-133a has been associated with tumorigenesis mechanisms and with diagnosis of several cancers in tissue samples [31,32]. However, its expression has been found heterogeneously altered in tumor tissue; for example, mir-133a is upregulated in cervical cancer [33] but downregulated in bladder cancer, squamous cell carcinoma, and metastatic prostate cancer [32,34,35]. Interestingly, miR-133a was found upregulated in the small airway epithelium of healthy smokers when compared with non-smokers [9], as well as in airway epithelial cells in a cigarette-smoke-exposure mouse model with airway hyper-responsiveness [36]. Experimentally, in vitro upregulation of miR-133a is related to the epithelial–mesenchymal transition of human airway epithelial cells [36] and lipid- and inflammatory signaling in human macrophages and endothelial cells [37]. Nevertheless, we could not find previous references of circulating miR-133a (in serum or plasma) associated with cigarette smoking or with lung adenocarcinoma diagnosis; therefore, our results indicated novel smoking-related miRNAs in the sera of AD patients that may be a non-invasive diagnostic biomarker for AD smokers. Our results also suggest a potential role of miR-133a-3p in the smoking-dependent mechanism of pathogenesis in lung cancer and in frequent lung cancer comorbidities, such as pulmonary hypertension, hypertensive disease, and cardiac disease. The role of miR-133a-3p should be experimentally tested in future studies to elucidate the biological mechanisms involved in lung cancer disease secondary to cigarette smoking.

We acknowledge some limitations of our study. First, the study sample size was relatively small and obtained from a single center. Second, we validated arbitrarily only five differentially expressed miRNAs. Third, experimental evidence of the molecular mechanisms involved in carcinogenesis or smoking-related lung pathogenesis was not provided. However, we consider that we obtained valuable information from a relatively small but well-characterized population of lung cancer patients in this first study. Moreover, although we only tested five miRNAs with the most significantly differential expression, the evidence obtained supports relevant findings that will direct the future research.

Finally, an ideal non-invasive biomarker miRNA should be able to discriminate lung cancer from non-cancer cases regardless of gender, ethnicity, and smoking status. However, cigarette smoking is associated with most lung cancer cases and is also related to pathogenesis mechanisms of lung carcinogenesis; therefore, the identification of those miRNAs that are associated with smoking represents a relevant, specific diagnostic tool for smokers and a strategy to elucidate the pathophysiological mechanisms of carcinogenesis related to smoking in the lung.

## 5. Conclusions

Our findings highlight how cigarette smoking affects the reliable identification of circulating miRNAs, miR-532-5p, miR-25-3p, and miR-133a-3p, as diagnostic biomarkers in lung cancer. Our data indicated that the three candidate miRNAs are associated with lung cancer-related mechanisms, but also suggested a smoking-dependent pathogenic role of miR-133a-3p in smokers.

## Figures and Tables

**Table 1:** Demographic and clinical characteristics of AD and healthy participants according to their cigarette-smoking status.

Characteristics	AD/CS−	AD/CS+	H/CS−	H/CS+
Discovery cohort				
Female, *n* (%)	15 (100)	15 (100)	15 (100)	15 (100)
Age, years, mean (SD)	62.8 (12.4)	68 (10)	57.9 (6.7)	56.6 (4.8)
BMI, kg/m^2^, mean (SD)	25 (5.2)	25 (3.4)	25.3 (2)	24.9 (3.2)
Cigarette smoking, *n* (%)	0 (0)	15 (100)	0 (0)	15 (100)
Pack/years, mean (SD)	NA	21.8 (7.9)	NA	37 (11.7) ‡
Diabetes mellitus, *n* (%)	2 (13)	0	0	0
Hypertension, *n* (%)	7 (46.7)	3 (20)	0	0
Cardiac disease, *n* (%)	1 (6.7)	2 (13.3)	0	0
Obesity	2 (13.3)	2 (13.3)	0	0
Histological subtype	AD	AD	NA	NA
Disease stage				
IIB, *n* (%)	5 (33.3)	4 (26.7)	NA	NA
IV, *n* (%)	10 (66.7)	11 (73.3)	NA	NA
Validation cohort				
Female, *n*	27 (100)	14 (100)	39 (100)	22 (100)
Age, years, mean (SD)	61.4 (12.3)	66.5 (10)	61 (8.2)	56 (5.9)
BMI, kg/m^2^, mean (SD)	25.1 (5.9)	25 (3.9)	26 (3.5)	26.5 (4.5)
Cigarette smoking, *n* (%)	0 (0)	14 (100)	0 (0)	29 (100)
Pack/years, mean (SD)	NA	22.2 (8.4)	NA	32.9 (13) ‡
Diabetes mellitus, *n* (%)	4 (14.8)	1 (7)	0	0
Hypertension, *n* (%)	5 (18.5)	2 (14.3)	0	0
Cardiac disease, *n* (%)	1 (3.7)	2 (14) §	0	0
Obesity	3 (11.1)	2 (14)	0	0
Histological subtype	AD	AD	NA	NA
Disease stage				
IIB	5 (18.5)	4 (28.6)	NA	NA
IIIB	4 (14.8)	2 (14.3)	NA	NA
IV	18 (66.7)	8 (57.1)	NA	NA

BMI, body mass index; AD, adenocarcinoma; CS, cigarette smoking; H, healthy control; n, sample size, NA, not applicable. Data are expressed as the mean ± standard deviation (SD). Differences between groups were assessed by using one-way ANOVA and Tukey’s post hoc test. Statistical significance *p* < 0.05. ‡ Statistical difference compared to AD/CS+. § Statistical difference compared to AD/CS−.

**Table 2:** Diagnostic accuracy values of miR-532-5p, miR-25-3p and miR-133a-3p as biomarkers for AD diagnosis according to the cigarette-smoking status.

MicroRNA	Study Groups	AUC	95% CI	Sensitivity (%)	Specificity (%)	*p*-Value
miR-532-5p	H vs. AD	0.745	0.641–0.849	76.5	59	<0.0001
	H/CS− vs. AD/CS−	0.762	0.649–0.875	81.5	56.4	<0.0001
	H/CS+ vs. AD/CS−	0.886	0.79–0.981	77.8	81.8	<0.0001
miR-25-3p	H vs. AD	0.674	0.565–0.782	76.5	60.7	0.005
	H/CS+ vs. AD/CS−	0.779	0.649–0.91	81.5	72.7	0.001
	H/CS+ vs. AD/CS+	0.779	0.578–0.98	85.7	72.7	0.028
miR-133a-3p	H vs. AD	0.588	NA	NA	NA	0.158
	H/CS+ vs. AD/CS+	0.935	0.836–1	85.7	95.5	0.001
miR-133a-3p	AD/CS− vs. AD/CS+	0.884	0.733–1	85.7	88.9	0.002
	H/CS− vs. H/CS+	0.765	0.627–0.902	82.1	68.2	0.001
miR-532-5p + miR-25-3p + miR-133a-3p	H vs. AD	0.70	0.596–0.804	70.6	59	0.001
miR-25-3p + miR-133a-3p	H/CS+ vs. AD/CS+	0.961	0.895–1	100	86.4	<0.0001

AD, adenocarcinoma; H, healthy; CS, cigarette smoking; AUC, area under the curve; CI, Confidence interval; NA, not applicable.

**Table 3:** Spearman correlation analysis for miRNA expression levels and continuous assessed variables.

Variable	miR-532-5p		miR-25-3p		miR-133a-3p	
	rho	*p*-Value	rho	*p*-Value	rho	*p*-Value
AD patients						
Age (years)	0.217	0.225	0.301	0.088	0.482 **	0.005
BMI (kg/m^2^)	0.162	0.377	−0.105	0.567	0.137	0.453
Pack/years	−0.352 *	0.041	−0.107	0.546	0.357 *	0.038
Healthy controls						
Age (years)	0.303 *	0.019	0.328 *	0.01	−0.036	0.783
BMI (kg/m^2^)	0.085	0.519	−0.043	0.746	−0.007	0.958
Pack/years	−0.283 *	0.027	−0.205	0.116	−0.45 **	<0.0001

AD, adenocarcinoma, BMI, body mass index. Statistically significant difference: *p* < 0.05 (* *p* < 0.05, ** *p* ≤ 0.01).

**Table 4:** Univariate and multivariate linear regression analysis of factors associated with miR-133a-3p levels in AD patients.

	Univariate Analysis			Multivariate Analysis		
Independent Factors	Coefficient B	95% CI	*p*-Value	Coefficient B	95% CI	*p*-Value
Age	0.311	−0.456–8.088	0.078			
BMI	0.731	−9.402–10.865	0.884			
Cigarette smoking status	145.048	47.162–242.933	0.005	5.009	−89.911–99.93	0.915
Pack/years	6.3	1.311–11.289	0.015	−19.101	−26.645–−11.556	<0.0001
Diabetes mellitus	−92.961	−246.252–60.331	0.226			
Hypertension	114.022	14.066–213.978	0.027			
Cardiac disease	197.707	34.312–361.103	0.019	33.62	−66.908–134.147	0.499
Obesity	91.719	−49.547–232.986	0.195			
ID group (AD/CS− vs. AD/CS+)	231.769	138.676–324.861	<0.0001	613.271	422.658–803.884	<0.0001

CI, confidence interval; BMI, body mass index; AD, adenocarcinoma; CS, cigarette smoking.

**Table 5:** Levels of miR-133a-3p were independently associated with the smoking status in AD patients in univariate linear regression analysis.

	**Univariate Analysis (Smoking as the Dependent Variable)**		
**Independent Factor**	**Coefficient B**	**95% CI**	***p*-Value**
miR-133a-3p	0.002	0–0.003	0.005
	**Univariate Analysis (Pack/Years as the Dependent Variable)**		
**Independent Factor**	**Coefficient B**	**95% CI**	***p*-Value**
miR-133a-3p	0.027	0.006–0.049	0.015

CI, confidence interval.

## Data Availability

Not applicable.

## Supplementary Materials

The following are available online at https://www.mdpi.com/article/10.3390/biology10080793/s1, Table S1: MiRNAs differentially expressed in serum from never-smoker AD patients (AD/CS−) compared to never-smoker healthy controls (H/CS−) in the discovery cohort, ranked by *p*-value. Table S2: MiRNAs differentially expressed in serum from smoker AD patients (AD/CS+) compared to smoker healthy controls (H/CS+) in the discovery cohort, ranked by *p*-value. Table S3: MiRNAs differentially expressed in serum from AD patients compared to healthy controls, including smokers and never-smokers, in the discovery cohort, ranked by *p*-value. Table S4: Complete results of the analysis of differentially expressed miRNAs in the screening phase using the TaqMan Human MicroRNA Array Panel v3.0, card A and card B. (a) AD/CS− vs. H/CS−; (b) AD/CS+ vs. H/CS+; (c) AD vs. H. Table S5: Diagnostic accuracy values of the combined biomarkers miR-532-5p, miR-25-3p, and miR-133a-3p for AD diagnosis. Table S6: Univariate linear regression analysis of factors associated with levels of miRNAs in AD patients. Table S7: Univariate linear regression analysis of factors associated with smoking status in AD patients. Table S8: Pathway enrichment analysis of miR-532-5p, miR-25-3p and miR-133a-3p. The table shows the enriched pathways with a significant *p* < 0.01 (KEGG database) or *p* < 0.05 (KEGG database), and *p* < 0.005 (DisGeNET database). The experimentally validated target genes of the candidate miRNAs were obtained from the miRTarBase v8.0 and TarBase v8.0 databases. Table S9: Experimentally validated genes that are common for more than two miRNAs and their associated enriched pathways.
